# Draft Genome Sequence of *Acinetobacter calcoaceticus* Strain GK1, a Hydrocarbon-Degrading Plant Growth-Promoting Rhizospheric Bacterium

**DOI:** 10.1128/genomeA.00909-15

**Published:** 2015-08-13

**Authors:** Panagiotis Gkorezis, Eric M. Bottos, Jonathan D. Van Hamme, Andrea Franzetti, Gennaro Roberto Abbamondi, Maria Balseiro-Romero, Nele Weyens, Francois Rineau, Jaco Vangronsveld

**Affiliations:** a Hasselt University, Centre for Environmental Sciences, Diepenbeek, Belgium; b Thompson Rivers University, Department of Biological Sciences, Kamloops, British Columbia, Canada; c University of Milano-Bicocca, Department of Environmental Sciences, Milano, Italy; d CNR, National Research Council of Italy, Institute of Biomolecular Chemistry, Pozzuoli, Naples, Italy; e University of Santiago de Compostela, Department of Soil Science and Agricultural Chemistry, Santiago de Compostela, Spain

## Abstract

The 3.94-Mb draft genome of *Acinetobacter calcoaceticus* GK1, a hydrocarbonoclastic plant growth-promoting Gram-negative rhizospheric bacterium, is presented here. Isolated at the Ford Motor Company site in Genk, Belgium, from poplar trees planted on a diesel-contaminated plume, GK1 is useful for enhancing hydrocarbon phytoremediation.

## GENOME ANNOUNCEMENT

The presence of *Acinetobacter* sp. strains in environments contaminated with pollutants such as diesel fuel, crude oil, phenol, and other recalcitrant organics has been well documented (1–5). *Acinetobacter calcoaceticus* GK1 was isolated from the rhizosphere of poplar trees in a diesel-contaminated environment. Phenotypic profiling and partial 16S rRNA gene sequence data showed that GK1’s closest relative is *Acinetobacter calcoaceticus* PHEA2 (GenBank accession no. CP002177).

Genomic DNA of GK1 was extracted with a Qiagen blood and tissue kit (Qiagen NV, Hilden, Germany) and an IonTorrent PGM was used to generate a whole-genome shotgun sequence using the methods described in reference 6.

A total of 371 Mb of data (>324 M Q20 bases) were generated in Torrent suite 4.2.1 and assembled into 35 contigs (uniform coverage mode; kmers 21, 33, 55, 77, 99) using SPAdes 3.1.0 (7, 8), giving a consensus length of 3,943,681 bp at 56× coverage (largest contig 587,330 bp; *N*_50_ = 228,328). Open reading frame (ORF) prediction and gene annotation was carried out using the PGAP (NCBI) pipeline (9). Contigs were ordered with the genome of *Acinetobacter calcoaceticus* PHEA-2 as a reference in Mauve (10).

The genome of *Acinetobacter calcoaceticus* GK1 consists of a single circular chromosome (39% G+C content), including 3,746 coding genes that were arranged into pathways using Pathway Tools (11, 12), 191 pseudogenes, 3 rRNAs (5S, 16S, 23S), 55 tRNAs, and 1 noncoding RNA (ncRNA).

Alkane-degradation genes were found spread across the genome, with homologues for 9 of the 12 *Acinetobacter calcoaceticus* PHEA2 genes located. Specifically, compared to PHEA2, GK1 has one additional copy of alk-1-monooxygenase (*alkB*), one fewer alcohol dehydrogenase (*alkJ*) gene, and one fewer methane monooxygenase gene. For aromatic hydrocarbons, most of the operon coding for the subunits of naphthalene dioxygenase is present with the loss of ferredoxin and reductase genes, the gain of a copy of the iron-sulfur subunit, loss of one copy of *nahB*, and the gain of one copy of *nahE*. Gentisate 1,2-dioxygenase (encoded by *nagI*; AOLE_09100) appears to be absent from the genomes of all sequenced *Acinetobacter* species, with the exception of strain DR1 (13). GK1 does possess two copies of a maleylacetoacetate isomerase gene similar to that found in DR1.

GK1 possesses an operon similar to the one that codes for production of the biosurfactant emulsan in other *Acinetobacter* spp., a trait that has utility for hydrocarbon biodegradation (14).

With respect to plant growth promotion, genes for 1-aminocyclopropane-1-carboxylate deaminase activity, auxin biosynthesis and organic phosphorous mineralization were located in the GK1 genome, in agreement with biochemical characterizations.

*Acinetobacter calcoaceticus* GK1 is being evaluated as an inoculant to enhance phytoremediation of diesel-contaminated sites.

### Nucleotide sequence accession numbers.

This whole-genome shotgun project has been deposited at DDBJ/EMBL/GenBank under the accession no. JYGV00000000. The version described in this paper is version JYGV01000000.
